# A Case Report: Post-Mortem Pathological Observations of a Fresh Dairy Cow with Type 3 Abomasal Ulcer After Sudden Death

**DOI:** 10.3390/ani15131969

**Published:** 2025-07-04

**Authors:** Greta Šertvytytė, Gabija Lembovičiūtė, Osvaldas Rodaitis, Karina Džermeikaitė, Samanta Arlauskaitė, Justina Krištolaitytė, Akvilė Girdauskaitė, Alius Pockevičius, Arūnas Rutkauskas, Ramūnas Antanaitis

**Affiliations:** 1 Large Animal Clinic, Veterinary Academy, Lithuanian University of Health Sciences, Tilžės Str. 18, LT-47181 Kaunas, Lithuania; gabija.lemboviciute@lsmu.lt (G.L.); osvaldas.rodaitis@stud.lsmu.lt (O.R.); karina.dzermeikaite@lsmu.lt (K.D.); samanta.arlauskaite@lsmu.lt (S.A.); justina.kristolaityte@lsmu.lt (J.K.); akvile.girdauskaite@lsmu.lt (A.G.); arunas.rutkauskas@lsmu.lt (A.R.); ramunas.antanaitis@lsmu.lt (R.A.); 2 Department of Veterinary Pathobiology, Veterinary Academy, Lithuanian University of Health Sciences, Tilžės Str. 18, LT-47181 Kaunas, Lithuania; alius.pockevicius@lsmu.lt

**Keywords:** type 3 perforated ulcer, abomasum, dairy cattle, sudden death, autopsy

## Abstract

In dairy cattle, abomasal ulcers are a serious but sometimes disregarded ailment that can have detrimental effects on health and cause financial losses. Due to inconclusive clinical symptoms, abomasal ulcers are typically misdiagnosed and treated improperly. Specialized diagnostic methods should be considered to ensure a correct diagnosis and the well-being of cattle. This report focuses on a 4-year-old Holstein-Friesian cow which began her third lactation two weeks before she started showing general clinical signs of an elevated fat–protein ratio in the milk and was diagnosed with an abomasum displacement. The clinical signs can also be mistaken for other conditions such as traumatic reticuloperitonitis and left dislocated abomasum. The patient was brought to the LUHS Large Animal Clinic, and after a short while, sudden death occurred. The autopsy concluded that death had occurred due to hypovolemic shock caused by abomasal ulcer perforation, which caused bleeding into the abomasum and intestines. Also, the type 3 ulcer caused severe peritonitis and anemia, and feed and fibrin could be seen on the outside of organs in the abdomen. Blood clots mixed with feed had formed in the inside of the abomasum and intestinal tract. Based on the work of previous scientific studies, it has been established that the occurrence of ulcers is more frequent in dairy cows during the first four to six weeks of lactation. And the most probable cause could be intensive feeding and dietary changes. Ulcers in the abomasum are very difficult to diagnose, because they require special diagnostic equipment such as an ultrasound or surgical interventions. Due to the similarity with other diseases, this pathological condition of the abomasum is most frequently only identified in post-mortem examinations.

## Simple Summary

Abomasal ulcers in dairy cows are serious but often overlooked due to vague symptoms that can be confused with other conditions. This report describes a 4-year-old Holstein-Friesian cow that died suddenly after showing general signs of illness. An autopsy revealed that the cause of death was a perforated type 3 abomasal ulcer, which led to internal bleeding, peritonitis, and anemia. These ulcers are most common in the first few weeks of lactation, likely due to diet changes and stress. Because their symptoms are unclear and require advanced tools for diagnosis, abomasal ulcers are often only discovered after death.

## 1. Introduction and Clinical Significance

Diagnostic confirmation of abomasal ulcers is usually obtained after slaughter or post-mortem, which poses significant barriers to timely therapeutic intervention [1].This reliance on post-mortem evaluation is suboptimal, especially in the veterinary environment, where early detection and management are crucial for animal welfare and productivity [2]. In this particular instance, the cow exhibited sudden and fatal symptoms following its transportation, and subsequent, a necropsy revealed a type 3 (according to Braun et al. [3]) ulcer and extensive hemorrhage in the abdominal cavity. With this case report, we aim to highlight the fundamental inaccuracies that were present in the initial diagnosis. The possible risks of abomasal ulcers should also be taken into consideration when managing or diagnosing a case of abomasum displacement in cattle. This is because the two conditions can occur simultaneously or present with similar clinical signs, so they should be carefully considered as part of the differential diagnoses to ensure thorough and accurate treatment planning [4].

## 2. Case Presentation

### 2.1. Handling

The dairy farm where the cow had been kept was located in the center of Lithuania. There were about 1200 milking cows on the farm. The barn was a free-stall setup, and Lely Astronaut 4 milking robots (Lely, Maassluis, The Netherlands) were used for robotic milking. The cows were fed a complete mixed ration every day at five o’clock in the morning and evening. It was balanced to fulfill the physiological and production requirements of a 550 kg Holstein-Friesian cow, which would typically produce 40 kg of milk per day. The composition of the total mix ration is presented in Table 1 and Table 2.

### 2.2. Medical History

**Figure 1:**
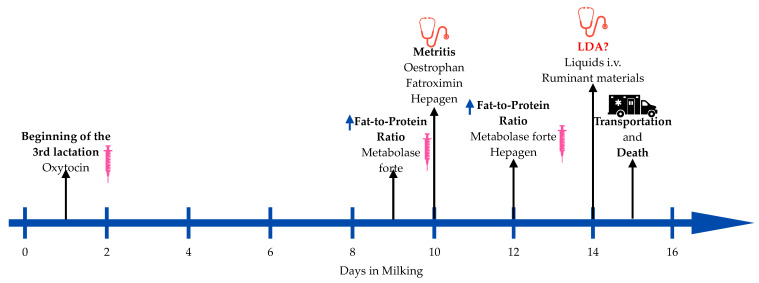
Cow’s health events 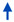 —an increase in the indicator. (figure created by authors with “Freeform” Version 3.5 (419.121.1)).

The cow was born on 07/05/2020; she began her third lactation on 4 September 2024, and was given OXYTOCIN BIOWET^®^ (oxytocin 10 TV/mL, Biowet Puławy Sp. z o.o, Puławy, Poland) 10 mL i.m. once for prevention of placenta retention. On the 9th day of milking, according to the automatic milking system (AMS) (Lely, Maassluis, The Netherlands), we identified that the cow had a higher than 1,4 fat-to-protein ratio in the milk. The cow’s weight, ruminating, milk production, fat-to-protein ratio, lactose, and eating changes in first 7 days of the third lactation are presented in Table 3. She was given Metabolase Forte^®^ (l-acetylmethionine 200 mg/mL, cyanocobalamin 0.2 mg/mL, l-carnitine hydrochloride 61.3 mg/mL, Fatro S. p. A., Ozzano dell′Emilia, Italy) 30 mL i.m. once. In the 10th DIM, the cow was diagnosed with metritis and treated with OESTROPHAN^®^ (cloprostenol 250 μg/mL, Bioveta a.s., Ivanovice na Hané, Czech Republic) 2 mL i.m., FATROXIMIN^®^ uterine foam (rifaximin—0.10 g/13.4 g of foam, Fatro S. p. A., Ozzano dell′Emilia, Italy), two units in the uterus, and HEPAGEN^®^ (phenoxy-2-methyl-2-propionic acid 100 mg/mL, Fatro S. p. A., Ozzano dell′Emilia, Italy) 40 mL i.m. At the 12th DIM, the fat–protein ratio increased again. This time, she received Metabolase Forte^®^ (l-acetylmethionine 200 mg/mL, cyanocobalamin 0.2 mg/mL, l-carnitine hydrochloride 61.3 mg/mL, Fatro S. p. A., Ozzano dell′Emilia, Italy) 30 mL i.m. and HEPAGEN^®^ (phenoxy-2-methyl-2-propionic acid 100 mg/mL, Fatro S. p. A., Ozzano dell′Emilia, Italy) 40 mL i.m. She was diagnosed with abomasum displacement to the left on the 14th DIM based on clinical indicators (positive fluctuation test, left-sided “ping” across the left flank of the cow). These tests might have come out positive due to other conditions, like air entering the abdominal cavity from the digestive tract through a perforated ulcer, as additional research showed [5]. Fluids were administered as rehydration therapy prior to surgery: RINGER LATATTO^®^ (lactic acid—1.3 g/500 mL, sodium hydroxide—0.575 g/500 mL, sodium chloride—3 g/500 mL, IZO srl a socio unico, Via San Zeno 99/A, 25124 Brescia, Italy), 1000 mL, METABOLASE^®^ (l-Carnitine hydrochloride 613.3 mg/500 mL, thioctic acid—20 mg/500 mL, pyridoxine hydrochloride—15 mg/500 mL, Fatro S. p. A., Ozzano dell′Emilia, Italy) 500 mL, GLUCOSE 25% SOLUTION WITH METHIONINE^®^ (glucose—125 g/500 g, methionine—19.5 g/500 mL IZO srl a socio unico, Via San Zeno 99/A, 25124 Brescia, Italy) 500 mL, and Calcibel Forte^®^ (calcium gluconate, injection 380 mg/mL, magnesium chloride hexahydrate 60 mg/mL, boric acid 50 mg/mL Bela-Pharm GmbH & Co. KG, Vechta, Germany) 120 mL s.c. Ruminative materials, RUMENGEL VET^®^ (glycerine, propylene glycol, magnesium sulfate, Over Group, Łask, Poland) 100 mL p.o., were also administered. The next day, she was sent to the Large Animal Clinic for abomasum displacement surgery, but 10–15 min. after transportation, the cow was discovered dead in the pen. We were unable to conduct further blood and diagnostic testing as a result. The cow’s health events timeline is presented in Figure 1.

### 2.3. Gross Lesions

The autopsy was performed three hours after death in the Lithuanian University of Health Sciences Veterinary Academy Pathology Center, and according to Braun et al. [3], this cow had type 3 abomasul ulcer.

**Figure 2:**
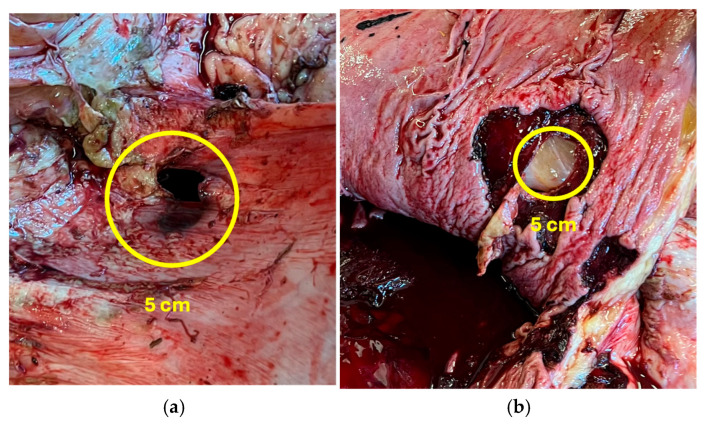
Perforated abomasal ulcer: (**a**) from the outside of the abomasum; (**b**) from the inside of the abomasum.

**Figure 3:**
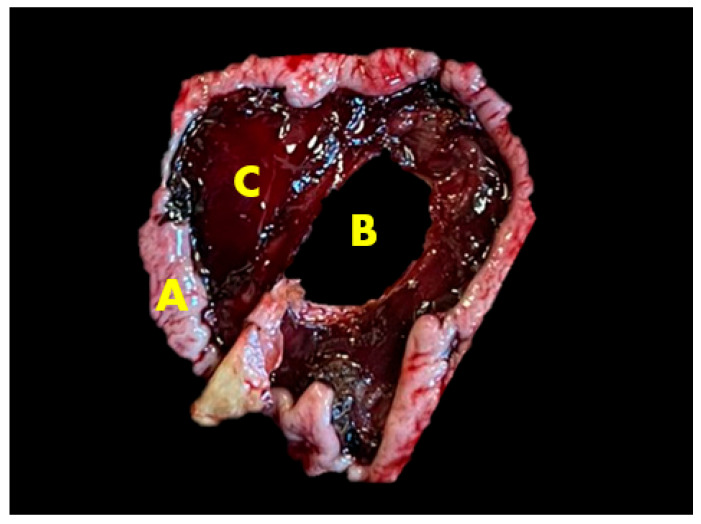
Perforated abomasal ulcer. A—Abomasal mucosa; B—ulcer hole; C—damaged and hemorrhagic abomasal mucosa.

The internal temperature of the cadaver was mildly decreased, and slight stiffness occurred when the autopsy began. The eyeballs were sunken into the eye sockets. The mucous membranes around the eyes and mouth were anemic, and slight dehydration in the subcutaneous tissue was found. The subcutaneous lymph nodes were enlarged with signs of edema, and the inside was gray and soft. A normal topographic position of organs in the abdominal cavity was determined, and there were no signs of abomasum displacement. The abomasum was found in the ventral part of the abdominal cavity, but there were visible ruminal contents on the outside of organs, and an unclear bloody inflammatory exudate was present in the abdomen. The spleen had shrunk, was stiff and unevenly colored, and had adhered to the peritoneum, while fibrin was found between the peritoneum and spleen parietal surface. Localized fibrinous peritonitis and feed were also visible on the outside of the stomach near the site of a perforated ulcer. Several other active ulcers were noticed on the inside of the abomasum, one of which perforated through the abomasal wall. The type 3 ulcer was located on the abomasal pyloric area, which was round shaped and measured at five centimeters in diameter. The perforated abomasal ulcer is presented in Figure 2 and Figure 3. The abomasum was filled with 10 L of clotted blood mixed with feed. The contents of the small and large intestine were greenish with bloody impurities. The lymph nodes of the intestine were enlarged and edematous.

Also, the milk glands of the cadaver had signs of inflammation, with swollen teats and viscous milk, which indicates mastitis. The uterus appeared inflamed, with a thickened uterine lining and redness, which suggests endometritis. An additional examination showed that hepatic lipidosis was present in the liver. While examining the chest cavity, the lungs presented hyperemia and emphysema, which may have been caused by the difficulties of breathing from the buildup of fluids in the abdomen. The heart was enlarged, with clotted blood in all chambers. The autopsy results concluded that the sudden death had occurred due to hypovolemic shock caused by the internal bleeding from the perforated ulcer and various complications caused by inflammation and accumulation of fluids in the abdomen. However, due to the rapid progression of this acute type 3 abomasal ulcer and an inaccurate initial diagnosis, there was insufficient time to collect blood and diagnostic samples from the cow.

## 3. Discussion

Several contributing factors may have affected the diagnostic accuracy and eventual outcome of this case. Firstly, a misdiagnosis occurred. As previously noted, no displacement was detected during the necropsy, suggesting that the tympanic sound heard during the on-farm clinical examination was more likely due to air in the gastrointestinal tract resulting from a perforated abomasal ulcer, rather than a left abomasum displacement.

Secondly, additional diagnostic tests were not performed. Due to the cow’s rapid deterioration and subsequent death shortly after arrival at the clinic, there was insufficient time for a comprehensive clinical workup, including blood sampling for hematological and biochemical analyses. Had the animal survived longer, further diagnostic procedures such as ultrasonography and a detailed clinical examination might have revealed signs that were indicative of an abomasal ulcer.

Moreover, no tissue samples were collected during necropsy. Such samples could have provided valuable insights into the concurrent clinical conditions—such as metritis, mastitis, and udder lesions—as well as potential underlying causes of the abomasal ulcer. The lack of tissue sampling not only limited the understanding of concurrent conditions but also hindered the identification of the specific type of abomasal ulcer present.

This distinction is clinically significant, as the symptoms can vary depending on the ulcer type. Clinical signs of type 3 abomasal ulcers are not specific, but they are similar to those of traumatic reticuloperitonitis and peritonitis. These include signs of abdominal pain, poor appetite, decreased ruminal motility, ruminal tympany, and enlarged abdomen. Additionally, the study carried out by Yasaswini et al. [6] presents a number of different clinical symptoms associated with abomasal ulcers in buffaloes. These include melena, a depressive behavior, tachycardia, an absence of rumination, abdominal guarding, kyphosis, and tachypnoea. Moreover, a thorough examination of the abomasum is essential to determine if it has been displaced. In a study conducted by Braun et al. [3], 35% of cattle tested positively on fluctuation and percussion auscultation tests on the left abomasum, and 32% on the right. In our study, the positive test result associated with abomasum displacement was identified because of localized peritonitis. As this pathological process of ulcers does not present specific clinical symptoms, it is necessary to assess the general condition of the animal and to carry out a comprehensive examination to provide the most accurate diagnosis. Nevertheless, a confirmed diagnosis of type 3 abomasal ulcer can frequently only be established through the performance of an exploratory laparotomy or a post-mortem examination.

One of the key clinical challenges in such cases is the interpretation of auscultation findings, particularly the presence of “ping” sounds, which can be misleading in the context of abomasal ulcers and peritonitis. The detection of left-sided “ping” sounds, elicited by simultaneous percussion and auscultation over the left flank of cattle, has proven to be an effective diagnostic tool for identifying left displaced abomasum (LDA) when combined with other physical examination techniques. Additionally, such sounds may also be present in cattle with other conditions, such as ruminal atony, ruminal tympany, and pneumoperitoneum, which complicates the diagnostic process. Therefore, it is crucial to carefully differentiate the “ping” sound to avoid misdiagnosis and ensure that other potential diseases are not overlooked. The misinterpretation of these sounds can lead to inaccurate conclusions, delaying appropriate treatment and potentially compromising animal health. A study by Gouda et al. demonstrated that while ultrasonographic examination of the “ping” area exhibited 100% sensitivity in detecting animals who were later confirmed to have LDA via exploratory laparotomy, its specificity was only 25%, leading to a substantial number of false-positive results. This highlights the importance of combining clinical findings with additional diagnostic tools to ensure a more accurate diagnosis and appropriate management of the affected animals [7,8]. The presence of clinical signs such as anorexia, weight loss, decreased ruminal motility, abdominal discomfort, and pain can present significant challenges in terms of differentiating LDA from peritonitis in dairy cattle [9]. It should be noted that peritonitis, which is often the result of traumatic reticuloperitonitis or abomasal ulceration, can produce gas–fluid interfaces that resemble the characteristic “ping” associated with LDA [7]. Furthermore, the presence of generalized inflammation can result in abnormal organ positioning and abdominal pain, which is a complicating factor in clinical assessment.

These diagnostic complexities are further compounded when systemic conditions, such as metritis and mastitis, are present, as seen in the current case. In the presented case study, metritis was detected, indicating a systemic inflammatory response. However, the exact time of development of the abomasal ulcer prior to death could not be determined, but it is likely that the ulcer and the metritis were related—the ulcer may have contributed to the development of the metritis, but it is unlikely that the metritis alone caused the ulcer. However, the combination of both conditions, along with associated metabolic stressors, may have led to the progression and severity of the ulcer. The co-existence of mastitis and endometritis in this particular case is indicative of a systemic inflammatory response, which is a well-documented contributor to metabolic dysfunction in early lactating dairy cows [10]. In addition, inflammatory mediators released during endometritis have been shown to disrupt endocrine and metabolic pathways, aggravating conditions such as abomasal ulcers and increasing the susceptibility to hypovolemic shock [11]. Overall, these findings support the hypothesis that mastitis and endometritis—beyond their local consequences—are part of a systemic syndrome that increases the susceptibility of transition cows to fatal complications.

Alongside systemic inflammation, metabolic indicators such as milk production and composition also offer valuable insights into the cow’s health status. The existing literature does not contain data regarding changes in milk composition in the presence of abomasal ulcers. However, in the current case, a decline in milk yield was observed on the sixth day postpartum, accompanied by a reduction in the fat/protein ratio on the fifth day of milking. A similar reduction in milk yield was observed in a study conducted by Hussain et al. [12], in which 78% of the lactating animals with suspected abomasal ulcer experienced a sudden decline in milk production. The abrupt decline in production can be explained by several factors, including the post-calving period, physiological changes, secondary health conditions and stress. The observed changes in the cow’s fat-to-protein ratio (FPR) are consistent with the metabolic stress patterns described in the literature. During the first seven days of lactation, the FPR increased steadily from 1.09 to 1.49 (see Table 3), thereby exceeding the critical threshold of 1.4 that is often associated with a negative energy balance (NEB) and early lactation metabolic disorders such as subclinical ketosis and displaced abomasum. This finding aligns with the observations reported by Cabezas-Garcia et al. [13], who established that an FPR above 1.4 in the early stages of lactation serves as a reliable indicator of NEB, particularly when the milk yield and body reserves have not yet been stabilized. Such shifts are indicative of metabolic strain, with a prolonged NEB having been linked to gastrointestinal complications, including abomasal ulcers [14]. The clinical outcome of this case—abomasal ulcer and sudden death—aligns with research describing how energy imbalances and metabolic stress in early lactation contribute to reduced gut integrity and immune suppression. Therefore, continuous monitoring of the FPR, particularly in high-producing cows during transition, offers a practical tool for the early detection of metabolic disorders and may inform timely interventions. Given the non-specific clinical signs and subtle metabolic shifts associated with abomasal ulcers, accurate and early diagnosis becomes essential to guide timely interventions and prevent fatal outcomes.

A comprehensive diagnostic examination is essential for the prevention of abomasal ulcer cases, as most of them remain subclinical, rarely detected during the early stages of development, and challenging to identify. In our case, type 3 perforated abomasal ulcer was diagnosed by a necropsy after a sudden death. The scientific literature recommends the implementation of ultrasound, diagnostic laparotomy, and a comprehensive post-mortem examination as diagnostic tests for pathological conditions. It is thought that ultrasound is an ineffective method for establishing the presence of ulcers. However, ultrasound examination is an adequate and useful method for visualizing the changes associated with perforated ulcers. Ultrasonography can be a valuable tool for evaluating the position, proportions, wall thickness, and content of the abomasum, as well as identifying possible inflammatory lesions that may affect adjacent organs [15]. Gerspach et al. [4] conducted a study which demonstrated that under certain circumstances, ultrasonographic imaging of a perforated abomasal ulcer in a cow is possible. The authors proposed that ultrasonography may be a viable alternative to exploratory surgery and a post-mortem examination for the antemortem diagnosis of a type 3 abomasal ulcer. The ultrasound examination in the study carried out by Braun et al. [3] revealed a presence of fibrin deposits in the serosa of the abomasum in five cows and free abdominal fluid in the abomasal region in six cows, which leads to a differential diagnosis of ulcers. Ultrasonography is a valuable diagnostic tool in the evaluation of cows with abomasum disorders, although the findings should be corroborated by other methods. Exploratory laparotomy is also a valuable diagnostic tool in ruminants, as it is relatively fast and inexpensive and provides a detailed examination of the abdominal cavity [16]. In the study carried out by Andrade et al. [17], exploratory laparotomy was performed in the region of the right para-lumbar fossa. The findings of the study revealed that in the presence of an abomasal ulcer, the abomasum was observed to be adhered to the peritoneum, dilated, and exhibiting focal peritonitis. Additionally, the formation of fibrosis and abscesses in the serosa was noted.

While imaging and surgical exploration provide valuable structural insights, histopathological analysis remains essential for understanding the underlying tissue-level pathology of abomasal ulcers. As previously mentioned, one of the main limitations in this case is the lack of histopathological sampling. Histopathology is a highly valuable diagnostic method for the definitive evaluation of gastrointestinal lesions observed during the autopsy. In a study by De Andrade Neto et al. [18] involving the histopathological examination of 201 abomasal ulcer fragments, the most common type 1 ulcer lesions included superficial necrosis of mucous cells with minor inflammation—sometimes presenting solely as the loss of mucus-secreting cells. Other findings included acute necrosis extending to the muscularis due to mucosal plate damage and chronic necrosis reaching the submucosa and muscular layers, forming three distinct zones: necrotic and inflammatory ulcer bed, granulation tissue, and dense fibrotic tissue with vascular changes in the submucosa. Some lesions also showed glandular atrophy with mild submucosal inflammation. In another study by the same author [17], which examined type 3, 4, and 5 ulcers, type 3 and 4 perforated ulcers were characterized by extensive necrosis with fibrosis, while type 5 ulcers exhibited widespread necrosis extending from the mucosa to the serosa, accompanied by the loss of glandular and vascular structures. Similarly, a study by Yasaswini et al. [19] identified mucosal infiltration by inflammatory cells, multifocal necrosis, degeneration, erosion, and loss of muscle architecture. However, the specific ulcer types were not stated in that study. Given the limited data available on the histopathological features of abomasal ulcers, future cases of this kind should include a histological examination as a standard diagnostic procedure to better understand the etiology and progression of slug ulcers.

Beyond diagnostic considerations, understanding the broader epidemiological and physiological context is equally important in assessing the development and progression of abomasal ulcers. Abomasal ulcers in dairy cattle remain insufficiently studied. Some research indicates a higher prevalence in the pre-calving and early post-calving periods, likely due to elevated cortisol levels and metabolic stress. Yasaswini et al. [19] reported a higher incidence of ulcers in buffaloes during early lactation. Similarly, Braun et al. [3] found that 57% of cows developed illness within four weeks postpartum. The acidic environment of the abomasum, exacerbated by high levels of volatile fatty acids from concentrated diets, contributes to mucosal damage and ulcer formation [20]. NSAIDs, while effective in managing inflammation, can negatively impact gastrointestinal and renal health due to COX-1 inhibition [21]. In this case, meloxicam and ketoprofen were administered, which may have influenced ulcer progression. Alternative treatments are emerging. For instance, Traumeel^®^ has shown pro-healing effects in stallions post-castration [22]. Bioactive peptides—derived from bone collagen, bovine prostate, or animal venoms—have demonstrated anti-inflammatory and analgesic properties [18,23,24]. These natural compounds hold promise as safer pain management options in veterinary care, although further research is necessary.

## 4. Conclusions

This example demonstrates the difficulties in diagnosing type 3 abomasal ulcers in dairy cows who are in the early stages of lactation. The overlapping clinical signs with other abdominal disorders, limitations in diagnostic workup, and absence of histopathological sampling hindered timely diagnosis and management. The abrupt mortality from ulcer perforation-induced hypovolemic shock highlights the drawbacks of depending only on post-mortem results. Because type III ulcers frequently result in unexpected mortality, this case report places a strong emphasis on practical application, highlighting the urgent need to improve diagnostic skills. Clinical evaluation is made more difficult by the fact that, for instance, the distinctive “ping” sound is not necessarily a trustworthy sign of abomasum displacement. The precise origin of the ulcer in this instance is still unknown, and the literature suggests a variety of potential contributing variables, making it challenging to identify a single etiology. We advise careful monitoring of a cow’s metabolic profile and vital signs during the transition phase, especially liver enzymes, non-esterified fatty acids (NEFAs), beta-hydroxybutyrate (BHB), and changes in milk output and fat-to-protein ratio, in order to enhance early identification. Furthermore, to improve clinical outcomes, a more integrated diagnostic approach—combining clinical examination, imaging, histopathology, and metabolic monitoring—is essential. Early, interdisciplinary assessment that incorporates imaging, metabolic tests, and clinical symptoms can enhance herd health management, facilitate prompt action, and lower the risk of catastrophic consequences. Continued research into alternative treatments and early biomarkers is also crucial to enhance prevention and intervention strategies for abomasal ulcers in high-risk dairy cows.

## Figures and Tables

**Table 1:** Composition of total mix ration.

Component of TMR *	%
Corn silage	24%
Grass hay	5%
Grass silage	16%
Grain concentrate slurry	50%
Mineral mixture	5%

* TMR—total mix ration.

**Table 2:** Chemical composition of total mix ration.

Parameters	%
DM Acid detergent fiber	48 20 (%/DM) *
Non-fiber carbohydrates	39 (%/DM)
Neutral detergent fiber	28 (%/DM)
Crude protein Net energy lactation	16 (%/DM) 1.6 (Mcal/kg) *

* DM—dry matter. %/DM—percents of dry matter per day. Mcal/kg—megacalories per kilogram of dry matter per day.

**Table 3:** Cow’s weight, ruminating, milk production, fat-to-protein ratio, lactose, and eating changes in first 7 days of 3rd lactation.

Date	Lactation Days	Rumination (min/24 h)	Milkings Per Day	Daily Production (kg)	Fat Content (%)	Protein Content (%)	Fat-to-Protein Ratio	Lactose Content (%)	Commonly Prescribed Feed (kg/Day)	Total Consumption (kg/Day)
4 September 2024	1	205	2	16.6	5.36	4.92	1.09	4.44	4.45	3.94
5 September 2024	2	263	4	24.7	5.36	4.57	1.17	4.57	4.71	4.94
6 September 2024	3	222	3	29.1	5.29	4.33	1.22	4.62	4.96	4.19
7 September 2024	4	246	3	28.4	5.43	4.1	1.32	4.65	5.22	5.54
8 September 2024	5	283	4	31.4	5.59	3.75	1.49	4.64	5.47	6.1
9 September 2024	6	165	2	30.3	5.52	3.75	1.47	4.64	5.73	3.67
10 September 2024	7	204	1	38.9	5.49	3.75	1.46	4.65	5.98	-

## Data Availability

Data is contained within the article.
